# Effects of transcranial magnetic stimulation in modulating cortical excitability in patients with stroke: a systematic review and meta-analysis

**DOI:** 10.1186/s12984-022-00999-4

**Published:** 2022-02-22

**Authors:** Zhongfei Bai, Jiaqi Zhang, Kenneth N. K. Fong

**Affiliations:** 1 Department of Rehabilitation Sciences, The Hong Kong Polytechnic University, Kowloon, Hong Kong SAR China; 2 Department of Occupational Therapy, Shanghai YangZhi Rehabilitation Hospital (Shanghai Sunshine Rehabilitation Center), Shanghai, China; 3 Department of Rehabilitation Sciences, Tongji University School of Medicine, Shanghai, China

**Keywords:** Stroke, Transcranial magnetic stimulation, Cortical excitability, Motor-evoked potentials, Interhemispheric imbalance

## Abstract

**Background:**

Transcranial magnetic stimulation (TMS) has attracted plenty of attention as it has been proved to be effective in facilitating motor recovery in patients with stroke. The aim of this study was to systematically review the effects of repetitive TMS (rTMS) and theta burst stimulation (TBS) protocols in modulating cortical excitability after stroke.

**Methods:**

A literature search was carried out using PubMed, Medline, EMBASE, CINAHL, and PEDro, to identify studies that investigated the effects of four rTMS protocols—low and high frequency rTMS, intermittent and continuous TBS, on TMS measures of cortical excitability in stroke. A random-effects model was used for all meta-analyses.

**Results:**

Sixty-one studies were included in the current review. Low frequency rTMS was effective in decreasing individuals’ resting motor threshold and increasing the motor-evoked potential of the non-stimulated M1 (affected M1), while opposite effects occurred in the stimulated M1 (unaffected M1). High frequency rTMS enhanced the cortical excitability of the affected M1 alone. Intermittent TBS also showed superior effects in rebalancing bilateral excitability through increasing and decreasing excitability within the affected and unaffected M1, respectively. Due to the limited number of studies found, the effects of continuous TBS remained inconclusive. Motor impairment was significantly correlated with various forms of TMS measures.

**Conclusions:**

Except for continuous TBS, it is evident that these protocols are effective in modulating cortical excitability in stroke. Current evidence does support the effects of inhibitory stimulation in enhancing the cortical excitability of the affected M1.

**Supplementary Information:**

The online version contains supplementary material available at 10.1186/s12984-022-00999-4.

## Background

Extensive investigations by means of transcranial magnetic stimulation (TMS) have provided pivotal insights into the cortical neurophysiology of patients who have suffered a stroke. Immediately after a stroke, impaired motor function accompanies substantial changes within the affected primary motor cortex (M1)—specifically, decreased corticospinal excitability, which can be reflected by the absence of recordable motor evoked potentials (MEPs)/decreased MEP amplitudes and increased resting/active motor thresholds (rMT/aMT) [1]. Besides, it is also evident that persistent disinhibition is detectable, regardless of chronicity [1], which is believed to have a facilitatory role in motor recovery [2]. For instance, the effects of rehabilitation training correlate with reduced intracortical inhibition, reflected by reduced short-interval intracortical inhibition (SICI) and long-interval intracortical inhibition [3, 4]. With respect to the cortical reorganization within the unaffected M1, as suggested by neuroimaging studies, the unaffected M1 in stroke patients became overactivated during movement execution of the affected hand [5]. Along with recovery of motor functions, stroke patients tend to regain the interhemispheric balance over the bilateral sensorimotor cortices [6]. However, meta-analyses found nonsignificant differences in a series of TMS measures, such as rMT, aMT and MEP amplitudes, compared to healthy controls [1].

In view of the association between cortical excitability and motor impairment of the hemiplegic arm, non-invasive brain modulation by repetitive TMS (rTMS) has attracted plenty of attention as it is effective in facilitating motor relearning and motor recovery after stroke. In accordance with the interhemispheric imbalance model, hemiparesis is caused not only by damaged corticospinal output from the affected M1, but also by excessive transcallosal inhibition from the unaffected to the affected M1 which can be measured by interhemispheric inhibition (IHI) and the ipsilateral silent period (iSP) [7]. Therefore, two unilateral modulatory approaches have been proposed—exciting or inhibiting the affected and unaffected M1—to counterbalance bilateral cortical excitability [8]. Early studies have revealed that both a single session of low frequency rTMS (LF-rTMS) and high frequency rTMS (HF-rTMS) were effective in improving the motor performance of the hemiparetic hand when they were applied to the unaffected and affected M1 [9, 10], respectively. From a neurophysiological perspective, LF-rTMS was shown to significantly reduce the MEPs of the unaffected M1 and the IHI from the unaffected to affected M1 [9, 10], while HF-rTMS had a direct facilitatory effect on the affected M1 [10].

Theta burst stimulation (TBS) is a unique form of rTMS that is usually delivered at subthreshold intensities over a short conditioning period [11, 12]. In healthy people, intermittent TBS (iTBS) was shown to enhance cortical excitability outlasting the stimulation period by almost 30 min, while opposite effects were shown after continuous TBS (cTBS) [13]. In stroke, iTBS to the affected M1 and cTBS to the unaffected M1 were shown to increase and decrease the MEPs, respectively [14]. Clinical studies suggested that both iTBS and cTBS were effective in modulating modulate the cortical excitability in patients with acute stroke [15]; iTBS, but not cTBS, was also able to change the cortical excitability in patients with chronic stroke [14, 16, 17].

Since the early introduction of TMS in the treatment of stroke [9], the clinical effects of various rTMS protocols have been well reviewed [18], and LF-rTMS to the unaffected M1 was shown to be the most effective form of treatment [19]. However, previous meta-analyses and reviews have generally focused only on clinical effects; effects from a neurophysiological perspective were less systematically and statistically reviewed [18]. Because of the inextricable relationship between cortical reorganization and motor recovery, it is necessary to consider how rTMS modulates cortical excitability, and whether the interhemispheric imbalance model is a valid hypothesis underlying the two therapeutic approaches. Therefore, the current systematic review and meta-analysis were conducted to evaluate the effects of four forms of rTMS (namely, LF-rTMS, HF-rTMS, iTBS, and cTBS) on a range of TMS measures of cortical excitability, including rMT, aMT, MEPs, SICI, intracortical facilitation (ICF), and iSP of bilateral M1s. Moreover, not only the accumulated effects of multiple sessions of rTMS, but also the effects of a single session of stimulation were evaluated. We also summarize the correlation between cortical excitability and motor improvement after multiple sessions of stimulation.

## Methods

This study was reported in accordance with the preferred reporting items for systematic reviews and meta-analysis statements [20].

### Search strategy

A systematic literature search was conducted by the first author using the following electronic databases: PubMed, Medline, EMBASE, CINAHL, and PEDro. The keyword combination of “((stroke) OR cerebrovascular accident) OR (hemiparesis) OR (hemiplegia)) AND ((transcranial magnetic stimulation) OR (TMS) OR (rTMS))” was used for the literature search. A manual search was also conducted, including screening the reference lists of previous systematic reviews and searching for the same keywords in Google Scholar. The last search was conducted on Jun 5, 2021.

### Selection criteria and data extraction

This study concerns the effects of both a single session and multiple sessions of rTMS. Therefore, the applied inclusion criteria were: (1) for studies investigating multiple sessions of rTMS, appropriate control groups must be employed; for studies investigating a single session of rTMS, a comparison between pre-rTMS and post-rTMS measures was indispensable; (2) studies using rTMS targeting the M1; (3) studies enrolling adult patients with a unilateral stroke; (4) studies having TMS measures of cortical excitability; (5) studies were published in English. Studies were excluded if they met any of the following exclusion criteria: (1) rTMS was applied in combination with other techniques; (2) bilateral TMS protocols; (3) studies in which necessary data regarding TMS measures were missing.

Once the study selection was completed, two authors independently extracted relevant data which were entered into two customized forms. Any discrepancies regarding data extraction were resolved through discussion.

### Quality assessment

A checklist proposed for critically appraising the quality of TMS procedures was used for all the studies [21]. Moreover, the Physiotherapy Evidence Database (PEDro) rating scale was used to appraise the general methodological quality of the studies with a parallel-group design [22].

### Data analysis

With respect to those studies ineligible for meta-analysis (e.g., skewed data), their main findings were qualitatively analyzed and integrated with the results of the meta-analyses. If there were two or less studies identified for a single analysis objective, we would not perform a meta-analysis but qualitatively described the results only. Since most studies investigating a single session of rTMS normalized the post-rTMS MEP amplitude to the pre-rTMS value, expressed as a percentage and SD, our meta-analyses computed a pooled mean of the percentage and its 95% confidence intervals (CI). Otherwise, absolute change scores were used to compute Hedges’ g, which corrected the possible bias of the small sample sizes. The Higgins’ I^2^ statistic was used to check heterogeneity across studies; an I^2^ value below 50% was considered to reflect low levels of heterogeneity, while an I^2^ value above 50% indicated high levels of heterogeneity. Given that the characteristics among the included studies were not exactly identical, a random-effects model was used for all the meta-analyses [23]. Publication bias was statistically examined using Egger’s linear regression test and by visually inspecting the funnel plot. The level of significance was set at p < 0.05 for all statistical analyses. Comprehensive Meta-analysis 3.0 software (Englewood, NJ, USA) was used to perform all the meta-analyses in the current study.

## Results

### Study characteristics

**Fig. 1:**
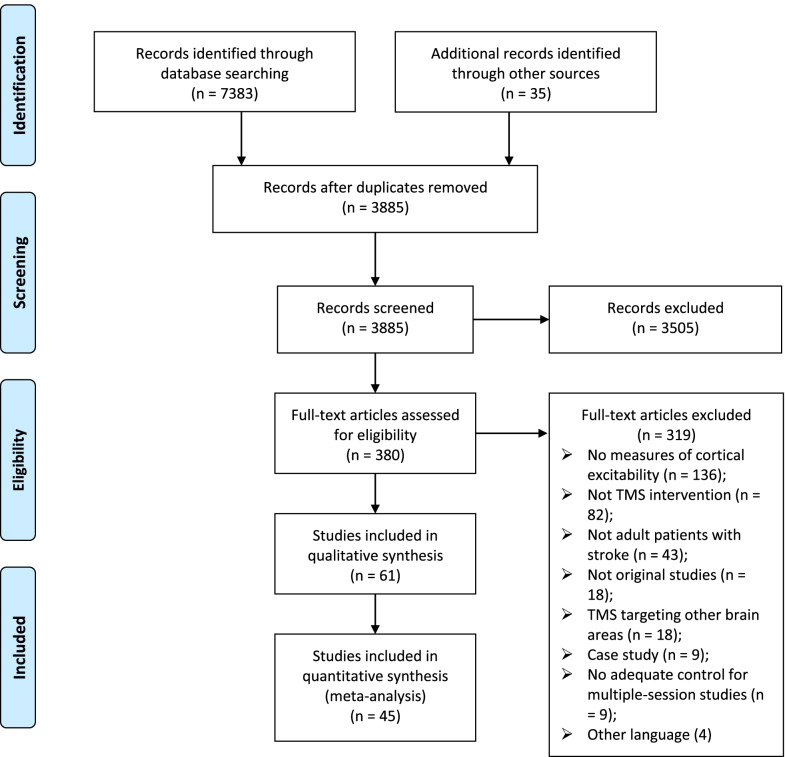
PRISMA flow diagram

The literature search process is presented in Fig. 1. Finally, 61 original studies were included, of which 45 studies were used for the meta-analyses. The characteristics of studies investigating a single session [9, 10, 14–17, 24–42] and multiple sessions [43–78] of rTMS are presented in Table 1 and Table 2, respectively.

**Table 1:** Characteristics of studies investigating the effects of a single session of rTMS

Study	n	Stroke	rTMS protocol	TMS measures
Takeuchi et al. (2005)	10	≥ 6 mth	Contra-FDI, 90% rMT, 1 Hz, 1500 pulses	UH: MEP; iSP (UH → AH)
Kim et al. (2006)	8	> 3 mth	Ipsi-FDI, 80% rMT, trains of 20 pulses at 10 Hz, ITI of 68 s, 160 pulses;	AH: MEP
Talelli et al. (2007)	6	≥ 1 year	Ipsi-FDI, 80% aMT, iTBS, 600 pulses Contra-FDI, 80% aMT, cTBS, 600 pulses	Bilateral: MEP;
Di Lazzaro et al. (2008)	12	≤ 10 days	Ipsi-FDI, 80% aMT, iTBS, 600 pulses Contra-FDI, 80% aMT, cTBS, 600 pulses	Bilateral: rMT, aMT, MEP
Takeuchi et al. (2008)	10	≥ 6 mth	Contra-FDI, 90% rMT, 1 Hz, 1500 pulses;	Bilateral: rMT; MEP; AH: SICI
Jayaram et al. (2009)	9	≥ 11 mth	Contra-low limb, 120% aMT, 1 Hz, 600 pulses	Bilateral: MEP
Takeuchi et al. (2009)	10	> 6 mth	(Conta-sham + Ipsi-FDI, 90% rMT, 10 Hz, 50 pulses) × 20	Bilateral: MEP; AH: SICI
Ackerley et al. (2010)	10	> 6 mth	Ipsi-FDI, 90% aMT, iTBS, 600 pulses Contra-FDI, 90% aMT, cTBS, 600 pulses	AH: MEP
Di Lazzaro et al. (2010)	17	< 10 days	Ipsi-FDI, 80% aMT, iTBS, 600 pulses	Bilateral: rMT, aMT, MEP
Takeuchi et al. (2012)	9	> 6 mth	Contra-FDI, 90% rMT, 1 Hz, 1200 pulses;	Bilateral: rMT, MEP; iSP (UH → AH), iSP (AH → UH)
Massie et al. (2013)	8	≥ 6 mth	Ipsi-FDI/APB, 70% rMT, trains of 30 pulses at 3 Hz, ITI of 30 s, 900 pulses	AH: rMT, SICI, ICF
Massie et al. (2013)	6	≥ 6 mth	Ipsi-FDI/APB, 70% rMT, trains of 30 pulses at 3 Hz, ITI of 30 s, 900 pulses	AH: SICI, ICF
Ackerley et al. (2014)	13	≥ 6 mth	Ipsi-FDI, 90% aMT, iTBS, 600 pulses Contra-FDI, 90% aMT, cTBS, 600 pulses	Bilateral: MEP
Vongvaivanichakul et al. (2014)	7	> 6 mth	Contra-APB, 90% rMT, 1 Hz, 1200 pulses	UH: MEP
Cassidy et al. (2015)	11	≥ 6 mth	Contra-Sham 6 Hz priming + Contra-1 Hz (FDI, 90% rMT, 1 Hz, 600 pulses)	AH: SICI, ICF, CSP; IHI (UH → AH), IHI ( AH → UH)
Goh et al. (2015)	10	> 6 mth	Ipsi-FDI, 90% r/aMT, trains of 50 pulses at 5 Hz, ITI of 30 s, 1200 pulses	AH: MEP
Tretriluxana et al. (2015)	9	> 6 mth	Contra-EDC, 90% rMT, 1 Hz, 1200 pulses	UH: MEP
Uhm et al. (2015)	16, 6	> 6 mth	Ipsi-FDI, 90% rMT, trains of 50 pulses at 10 Hz, ITI of 55 s, 1000 pulses Ipsi-FDI, 110% rMT, trains of 50 pulses at 10 Hz, ITI of 55 s, 1000 pulses	AH: MEP
Bashir et al. (2016)	8	> 6 mth	Contra-FDI, 90% rMT, 1 Hz, 1200 pulses	Bilateral: rMT, MEP, CSP, SICI, ICF
Di Lazzaro et al. (2016)	15, 20	< 10 days	Ipsi-FDI, 80% aMT, iTBS, 600 pulses	Bilateral: rMT, aMT, MEP
Murdoch et al. (2016)	12	≥ 6 mth	Ipsi-FDI, 80% aMT, iTBS, 600 pulses	AH: MEP, SICI
Diekhoff-Krebs et al. (2017	14	≥ 12 mth	Ipsi-FDI, 80% aMT, iTBS, 600 pulses	Bilateral: MEP
Khan et al. (2017)	15	< 3 mth	Ipsi-FDI, 60% rMT, iTBS, 600 pulses	AH: rMT, MEP, CSP
Hanafi et al. (2018)	8	≥ 6 mth	Contra-FDI, 90% rMT, 1 Hz, 1200 pulses Ipsi-FDI, 80% rMT, trains of 50 pulses at 10 Hz, ITI of 25 s, 1000 pulses	Bilateral: MEP
Tretriluxana et al. (2018)	8	1—6 mth	Contra-EDC, 90% rMT, 1 Hz, 1200 pulses	UH: MEP

*TMS* = transcranial magnetic stimulation, *rTMS* = repetative TMS, *mth* months, *Contra* contralesional, *Ipsi* ipsilesional, *FDI* first dorsal interosseous, *UH* unaffected hemisphere, *AH* affected hemisphere, *MEP* motor-evoked potential, *iSP* iplilateral slient period, *ITI* inter-train interval, *iTBS* intermittent theta burst stimulation, *cTBS* continuous theta burst stimulation, *aMT*, active motor threshold, *rMT* resting motor threshold, *SICI* short interval intracortical inhibition, *APB* abductorr pollicis brevis, *ICF* intracortical facilitation, CSP cortical slient period, *IHI* interhemisperic inhibition, *EDC* extensor digitorum communis

**Table 2:** Characteristics of studies investigating the effects of multiple sessions of rTMS

Study	N (E, C)		Chronicity	E intervention	C intervention	rTMS protocol	TMS measures
Khedr et al. (2005)	26	26	Acute	Ipsi-rTMS + Conv	Ipsi-Sham rTMS + Conv	ADM, 120% rMT, trains of 30 pulses at 3 Hz, ITI of 50 s, 300 pulses, 10 sessions	AH: MEP presented or not
Fregni et al. (2006)	10	5	> 1 yr	Contra-rTMS	Contra-Sham rTMS	FDI, 100% rMT, 1 Hz, 1200 pulses, 5 sessions	Bilateral: rMT
Malcolm et al. (2007)	9	10	≥ 1 yr	Ipsi-rTMS + CIMT	Ipsi-Sham rTMS + CIMT	FDI, 90% rMT, trains of 40 pulses at 20 Hz, ITI of 28 s, 2000 pulses, 10 sessions	AH: rMT
Pomeroy et al. (2007)	6	7	1—12 wk	Ipsi-rTMS + VMC	Ipsi-Sham rTMS + VMC	Triceps, 120% rMT, trains of 40 pulses at 1 Hz, ITI of 3 min, 200 pulses, 8 sessions	MEP frequency
	4	7		Ipsi-rTMS + Sham-VMC	Ipsi-Sham rTMS + Sham-VMC		
Khedr et al. (2009)	12	12	7—20 days	Contra-rTMS	Ipsi-Sham rTMS	Contra: FDI, 100% rMT, 1 Hz, 900 pulses, 5 sessions	Bilateral: aMT, MEP
	12			Ipsi-rTMS		Ipsi: FDI, 130% rMT, trains of 30 pulses at 3 Hz, ITI of 2 s, 900 pulses, 5 sessions	
Khedr et al. (2010)	9	8	5—15 days	Ipsi-rTMS (3 Hz)	Ipsi-Sham rTMS	3 Hz: FDI, 130% rMT, trains of 15 pulses at 3 Hz, 750 pulses, 5 sessions	Bilateral: rMT, aMT, MEP
	9			Ipsi-rTMS (10 Hz)		10 Hz: FDI, 100% rMT, trains of 20 pulses at 10 Hz, 750 pulses, 5 sessions	
Theilig et al. (2011)	12	12	2 w—58 mth	FNMS + Contra-rTMS	FNMS + Contra-sham rTMS	FDI, 100% rMT, 1 Hz, 900 pulses, 10 sessions	UH: MEP
Avenanti et al. (2012)	8	14	> 6 mth	Contra-rTMS + PT	Contra-Sham rTMS + PT	FDI, 90% rMT, 1 Hz, 1500 pulses, 10 sessions	Bilateral: rMT; iSP (UH → AH)
Wang et al. (2012)	12	12	> 6 mth	Contra-rTMS + Trask training	Contra-Sham rTMS + Task training	Rectus femoris, 90% rMT, 1 Hz, 600 pulses, 10 sessions	Bilateral: MEP
Di Lazzaro et al. (2013)	4	4	≥ 1 yr	Contra-cTBS + PT	Contra-Sham cTBS + PT	FDI, 80% aMT, cTBS, 600 pulses, 10 sessions	Bilateral: rMT, aMT, MEP
Hsu et al. (2013)	6	6	2—4 wk	Ipsi-iTBS + conv	Ipsi-Sham iTBS + conv	APB/ECR, 80% aMT, iTBS, 1200 pulses, 10 sessions	Bilateral: aMT; AH: MEP
Sung et al. (2013)	15	14	3—12 mth	Contra-rTMS + ipsi-iTBS	Contra-Sham rTMS + ipsi-Sham	rTMS: FDI, 90% rMT, 1 Hz, 600 pulses, 10 sessions iTBS: FDI, 80% aMT, iTBS, 600 pulses, 10 sessions	Bilateral: rMT, MEP
	12			Contra-Sham rTMS + ipsi-iTBS			
	13			Contra-rTMS + ipsi-Sham iTBS			
Rose et al. (2014)	9	10	> 6 mth	Contra-rTMS + Trask training	Contra-Sham rTMS + Trask training	ECR, 100% rMT, 1 Hz, 1200 pulses, 16 sessions	UH: rMT, SICI
Wang et al. (2014a)	16	14	3—12 mth	Contra-rTMS	Contra-Sham rTMS	FDI, 90% rMT, 1 Hz, 600 pulses, 10 sessions	Bilateral: rMT, MEP
Wang et al. (2014b)	17	16	2—6 mth	Contra-rTMS + ipsi-iTBS	Contra-Sham rTMS + ipsi-Sham iTBS	rTMS: FDI, 90% rMT, 1 Hz, 600 pulses, 10 sessions iTBS: FDI, 80% aMT, iTBS, 600 pulses, 10 sessions	Bilateral: rMT, MEP
	15			Ipsi-iTBS + Contra-rTMS			
Blesneag et al. (2015)	8	8	10 days	Contra-rTMS	Contra-Sham rTMS	APB, 120% rMT, 1 Hz, 1200 pulses, 10 sessions	Bilateral: rMT
Ludemann-Podubecka et al. (2015)	20	20	< 6 mth	Contra-rTMS + standard treatment	Contra-Sham rTMS + standard treatment	FDI, 100% rMT, 1 Hz, 900 pulses, 15 sessions	UH: MEP
Mello et al. (2015)	9	9	5—45 days	Contra-rTMS	Contra-Sham rTMS	APB, 90% rMT, 1 Hz, 1500 pulses, 10 sessions	UH: rMT, SICI, ICF
Srikumari et al. (2015)	30	30	10 d—1 mth	Ipsi-rTMS + conv	Ipsi-Sham rTMS + conv	APB, 80% rMT, trains of 20 pulses at 10 Hz, ITI of 58 s, 160 pulses, 5 sessions	AH: rMT
Du et al. (2016a)	15	16	3—30 d days	Ipsi-rTMS + PT	Contra-Sham rTMS + PT	Ipsi: APB, 80—90% rMT, trains of 30 pulses at 3 Hz, ITI of 10 s, 1200 pulses, 5 sessions	Bilateral: rMT, MEP
	14			Contra-rTMS + PT		Contra: APB, 110—120% rMT, trains of 30 pulses at 1 Hz, ITI of 2 s, 1200 pulses, 5 sessions	
Du et al. (2016b)	12	10	< 2 mth	Ipsi-rTMS + conv	Contra-Sham rTMS + conv	Ipsi: mylohyoid, 90% rMT, trains of 30 pulses at 3 Hz, ITI of 10 s, 1200 pulses, 5 sessions	Bilateral: MEP
	9			Contra-rTMS + conv		Contra: mylohyoid, 100% rMT, trains of 30 pulses at 1 Hz, ITI of 2 s, 1200 pulses, 5 sessions	
Volz et al. (2016)	13	13	< 2 wk	Ipsi-iTBS (APB, M1) + PT	Ipsi-iTBS (parieto-occipital vertex) + PT	70% aMT, iTBS, 600 pulses, 10 sessions	AH: rMT, MEP
Cha et al. (2017)	10	10	< 6 mth	rTMS + exercise	Excise	Soleus, 90% rMT, trains of 100 pulses at 1 Hz, ITI of 2 s, 1000 pulses, 40 sessions	AH: MEP
Guan et al. (2017)	13	14	< 1 wk	Ipsi-rTMS	Ipsi-sham rTMS	ADM, 120% rMT, trains of 20 pulses at 5 Hz, ITI of 2 s, 1000 pulses, 10 sessions	AH: rMT
Huang et al. (2018)	18	20	10—90 days	Contra-rTMS + PT	Contra-sham rTMS + PT	Rectus femoris, 120% aMT, 1 Hz, 900 pulses, 15 sessions	UH: aMT, MEP
Watanabe et al. (2018)	8	6	< 7 days	Ipsi-iTBS + PT + OT	Ipsi-sham iTBS + PT + OT	FDI, 80% rMT, iTBS, 600 pulses, 10 sessions	AH: MEP
	7			Contra-rTMS + PT + OT		FDI, 110% aMT, 1 Hz, 1200 pulses, 10 sessions	
Dos Santos et al. (2019)	10	10	≥ 6 mth	Contra-rTMS + PT	Contra-sham rTMS + PT	FDI, 110% rMT, 1 Hz, 1500 pulses, 10 sessions	UH: intensity inducing MEPs of 1 mv
Du et al. (2019)	12	12	< 2 wk	Ipsi-rTMS + PT	Contra-Sham rTMS + PT	Ipsi: APB, 100% rMT, trains of 40 pulses at 10 Hz, ITI of 40 s, 1200 pulses, 5 sessions	Bilateral: rMT, MEP
	12			Contra-rTMS + PT		Contra: APB, 100% rMT, trains of 120 pulses at 1 Hz, ITI of 40 s, 1200 pulses, 5 sessions	
El-Tamawy et al. (2019)	20	20	4.4—4.5 mth	Contra-rTMS + PT	PT	FDI, 90% aMT, 1 Hz, 1200 pulses, 10 sessions	Bilateral: aMT
Neva et al. (2019)	12	12	≥ 6 mth	Contra-cTBS	Contra-sham cTBS	Contra-ECR, 80% cTBS, 600 pulses	Bilateral: rMT, aMT, SICI, ICF; iSP (UH → AH), iSP (AH → UH)
Wang et al. (2019)	8	6	> 6 mth	Ipsi-rTMS + treadmill training	Ipsi-sham rTMS + treadmill training	Tibialis anterior, 90% rMT, trains of 60 pulses at 5 Hz, ITI of 48 s, 900 pulses, 9 sessions	Bilateral: MEP
Zhang et al. (2019)	16	14	< 2 mth	Ipsi-rTMS + NMES	Ipsi-sham rTMS + NMES	Ipsi: mylohyoid, 110% rMT, trains of 30 pulses at 3 Hz, ITI of 27 s, 900 pulses, 10 sessions	Bilateral: MEP
	15			Contra-rTMS + NMES		Contra: mylohyoid, 80% rMT, 1 Hz, 900 pulses, 10 sessions	
Wang et al. (2020)	15	15	2 w to 3 mth	Contra-HF-rTMS + PT + OT	Contra-sham HF-rTMS	APB, 100% rMT, trains of 10 pulses at 10 Hz, ITI of 10 s, 1000 pulses, 10 sessions	UH: MEP
	15			Contra-LF-rTMS + PT + OT		APB, 100% rMT, trains of 10 pulses at 1 Hz, ITI of 3 s, 1000 pulses, 10 sessions	
Hassan et al. (2020)	12	8	5.76 (0.55) mth	Ipsi-rTMS	Sham-rTMS	FDI, 80% rMT, trains of 50 pulses at 10 Hz, ITI of 25 s, 1000 pulses, 10 sessions	AH: MEP
	9			Contra-rTMS		FDI, 90% rMT, 1 Hz, 1200 pulses, 10 sessions	
Ke et al. (2020)	16	16	< 2 wk	Ipsi-rTMS + conv	Sham-rTMS + conv	ABP, 110% rMT, trains of 40 pulses at 20 Hz, ITI of 8 s, 1200 pulses, 10 sessions	AH: MEP
	16			Ipsi-rTMS + conv		ABP, 110% rMT, trains of 40 pulses at 20 Hz, ITI of 28 s, 1200 pulses, 10 sessions	
Gong et al. (2021)	16	16	< 30 days	Contra-rTMS	Sham	Trains of 40 pulses at 1 Hz, ITI of 2 s, 1200 pulses, 10 sessions	Bilateral: rMT, MEP

*E* experimental group, *C* control group, *wk* weeks, *mth* months, *yr* years, *TMS* transcranial magnetic stimulation, *rTMS* repetative TMS, *Conv* conventional treatment, *MEP* motor-evoked potential, *Ipsi* ipsil, *ADM* abductor digiti minimi, *ITI* inter-train interval, *AH* affected hemisphere, Contra contralesional, *FDI* first dorsal interosseous, *rMT* resting motor threshold, *CIMT* constraint-induced movement therapy, *VMC* voluntary muscle contraction, *aMT*, active motor threshold, *FNMS* functional neuromuscular stimulation, *UH* unaffected hemisphere, *PT* physical therapy, *cTBS* continuous theta burst stimulation, *iSP* iplilateral slient period, *iTBS* intermittent theta burst stimulation, *SICI* short interval intracortical inhibition, *APB* abductor pollicis brevis, *ECR* extensor carpi radialis, *ICF* intracortical facilitation, *M1* primary motor cortex, *OT* occupational therapy, *HF* high frequency, *LF* low frequency

The methodological quality of the TMS procedures is presented in Supporting Information (Additional file 1: Tables S1 and S2). Overall, the characteristics of the participants, coil type, and intensity were well reported. However, medication, CNS drugs, coil orientation, current direction, pulse shape, time interval between MEPs, and subject attention were missing in most studies. In addition, only a few studies used a navigation system to monitor the real-time position of coils in relation to the head of the participants. The PEDro score indicated that the included studies with a parallel-group design had good general methodological quality (Table 3), with a mean score of 7.2 (SD = 1.18). The funnel plots used in the publication bias examination are presented in Supporting Information (Additional file 1: Figs. S1 to S17).

**Table 3:** PEDro scores of the included studies with a parallel-group design

Authors	PEDro items											Total
	1	2	3	4	5	6	7	8	9	10	11	
Khedr et al. (2005)	1	1	0	1	1	0	1	1	1	1	1	8
Fregni et al. (2006)	1	1	0	1	0	0	0	1	1	0	1	5
Malcolm et al. (2007)	1	1	0	0	1	1	0	1	1	1	1	7
Pomeroy et al. (2007)	1	1	1	1	0	0	1	1	1	0	1	7
Khedr et al. (2009)	1	1	0	1	1	0	1	1	1	0	1	7
Khedr et al. (2010)	1	1	0	1	1	0	1	1	0	1	1	7
Theilig et al. (2011)	1	1	0	1	0	0	1	1	1	1	1	7
Avenanti et al. (2012)	1	1	0	1	1	1	1	1	1	1	1	9
Wang et al. (2012)	1	1	1	1	1	0	1	1	0	1	1	9
Di Lazzaro et al. (2013)	1	1	0	1	1	1	0	1	1	1	1	8
Hsu et al. (2013)	1	1	0	1	1	0	1	1	1	1	1	8
Sung et al. (2013)	1	1	1	1	0	1	1	1	1	1	1	9
Rose et al. (2014)	1	1	0	0	0	1	0	1	0	1	1	6
Wang et al. (2014a)	1	1	0	1	0	1	0	1	1	1	1	7
Wang et al. (2014b)	1	1	1	1	0	1	1	1	1	1	1	9
Blesneag et al. (2015)	1	1	0	0	0	0	1	1	1	1	1	6
Ludemann-Podubecka et al. (2015)	1	1	1	1	1	0	1	1	1	1	1	9
Mello et al. (2015)	1	1	0	1	1	0	0	1	1	1	1	7
Srikumari et al. (2015)	1	1	0	0	0	0	0	1	1	1	1	5
Du et al. (2016a)	1	1	1	1	1	0	1	1	0	1	1	8
Du et al. (2016b)	1	1	1	1	1	1	1	1	0	1	1	9
Volz et al. (2016)	1	0	0	1	0	0	1	1	1	1	1	6
Cha et al. (2017)	1	1	1	1	1	0	1	1	1	1	1	9
Guan et al. (2017)	1	1	1	1	0	1	1	0	0	1	1	7
Huang et al. (2018)	1	1	0	1	1	0	1	1	1	1	1	8
Watanabe et al. (2018)	1	1	1	1	0	0	1	1	1	1	1	8
Dos Santos et al. (2019)	1	1	1	1	0	1	1	1	0	1	1	8
Du et al. (2019)	1	1	1	1	1	1	1	1	0	1	1	9
El-Tamawy et al. (2019)	1	1	0	1	0	0	1	1	1	1	1	7
Neva et al. (2019)	1	0	0	1	1	0	0	1	0	1	1	5
Wang et al. (2019)	1	1	0	1	1	0	1	1	1	1	1	8
Zhang et al. (2019)	1	1	0	1	0	0	1	1	0	1	1	7
Wang et al. (2020)	1	1	0	1	0	0	1	1	1	1	1	7
Hassan et al.(2020)	1	1	1	1	0	0	0	1	1	1	1	7
Ke et al. (2020)	1	1	0	1	0	0	1	1	1	1	1	7
Gong et al. (2021)	1	1	1	1	0	0	1	1	0	1	1	7

1 = eligibility criteria. 2 = random allocation. 3 = concealed allocation. 4 = baseline comparability. 5 = blind subjects. 6 = blind therapists. 7 = blind assessors. 8 = adequate follow-up. 9 = intention-to-treat analysis. 10 = between-group comparisons. 11 = point estimates and variability

### Low frequency rTMS to the unaffected M1

Pomeroy et al. applied one Hz rTMS to the affected M1, rather than to the unaffected M1, and found that MEP responses of the hemiparetic arm were more frequently evoked after receiving real rTMS than sham rTMS [46]. Recently, Wang et al. found that HF-rTMS applied to the unaffected M1 yielded greater improvement on the MEP amplitude than LF-rTMS, suggesting a compensatory role of the unaffected M1 in the motor recovery of severe-impaired patients [75].

The effects after a single session and multiple sessions of LF-rTMS were evaluated by 11 [9, 24–26, 28, 31, 32, 34, 36, 41, 42] and 24 [44, 46, 47, 49–51, 54–60, 62, 63, 65, 67–71, 74, 76, 78] studies, respectively. Dos Santos et al. found that 10 sessions of LF-rTMS increased the cortical excitability of the unaffected M1, measured using an intensity tracking measuring approach that the intensity inducing MEPs of 1 mV by a single-pulse TMS stimulation was determined in the study to represent corticospinal excitability [69]. Moreover, LF-rTMS was found to be effective in rebalancing bilateral excitability by increasing the MEP amplitude of the affected M1 and decreasing the MEP amplitude of the unaffected M1 [56].

#### Resting/active motor threshold

**Fig. 2:**
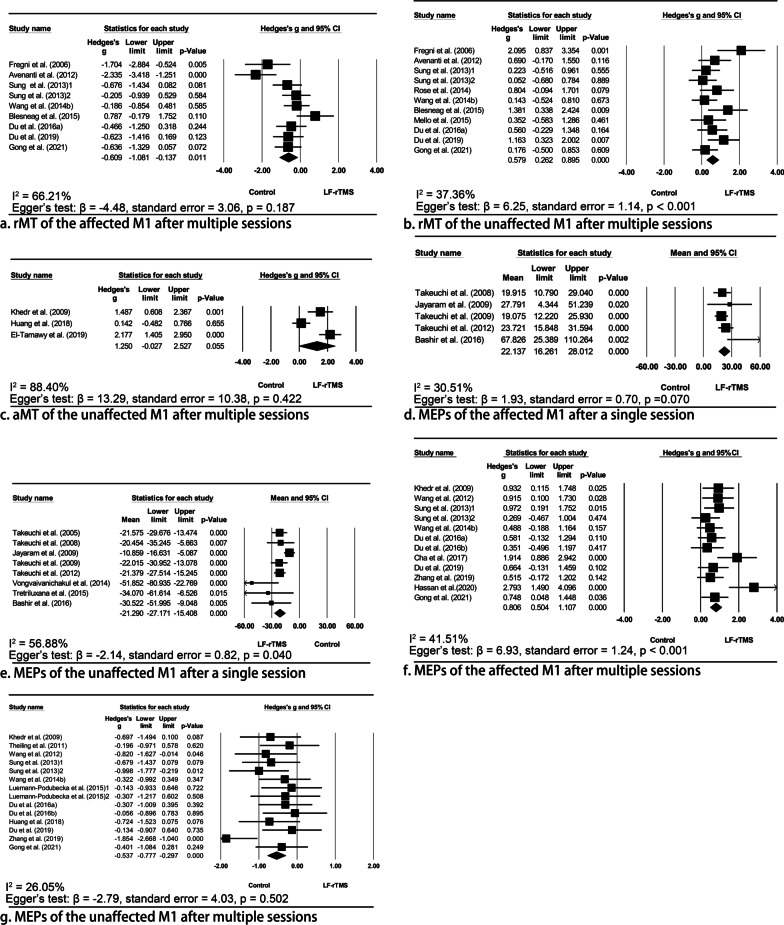
Meta-analyses indicating the effects of low frequency repetitive transcranial magnetic stimulation (LF-rTMS) to the unaffected M1 in modulating bilateral cortical excitability. The meta-analysis showed that LF-rTMS was significantly effective to decrease (**a**) and increase (**b**) rMT of the affected and unaffected M1 after multiple sessions of stimulation, respectively. Although LF-rTMS also tended to increase the aMT of the unaffected M1, the pooled Hedges’ g value was not significant (**c**). A single session of LF-rTMS significantly increased the MEP amplitude of the affected M1 by 22.14% (**d**); similar results were also found after multiple sessions of stimulation (**f**). Conversely, the MEP amplitude of the unaffected M1 significantly decreased by 21.29% immediately after a single session of LF-rTMS (**e**); similarly, the MEP amplitude of the unaffected M1 significantly decreased after multiple sessions of LF-rTMS (**g**). *rMT* resting motor threshold; *aMT* active motor threshold, *MEP* motor-evoked potentials

Previously, significant modulatory effects on rMT induced by a single session of LF-rTMS were not found [24, 28, 36]. However, our meta-analysis has shown the significant effect of multiple sessions of LF-rTMS in decreasing the rMT of the affected M1 (Hedges’ g = − 0.61, 95% CI = − 1.08 to − 0.14, p = 0.011, I^2^ = 66.21%; Fig. 2), and no publication bias was identified (p = 0.187). It suggested that the decreased rMT could be sustained for three months after the intervention [50, 62], while no significant long-term effects were also reported [58]. Likewise, multiple sessions of LF-rTMS also decreased the aMT of the affected M1 immediately [47, 71].

Conversely, the rMT of the unaffected M1 significantly increased after multiple sessions of LF-rTMS (Hedges’ g = 0.579, 95% CI = 0.26—0.90, p < 0.001, I^2^ = 37.36%), but significant publication bias was identified (p < 0.001). Inconsistent findings were reported with respect to its long-term effects [50, 55, 58, 62]. Although LF-rTMS also tended to increase the aMT of the unaffected M1, the pooled Hedges’ g value was not significant (Hedges’ g = 1.25, 95% CI = − 0.03–2.53, p = 0.055, I^2^ = 88.40%); the publications bias was not significant (p = 0.422).

#### Amplitude of motor-evoked potentials

A single session of LF-rTMS significantly increased the MEP amplitude of the affected M1 by 22.14% (95% CI = 16.26%—28.01%, p < 0.001, I^2^ = 30.51%) and no publication bias was identified (p = 0.070). Similar results were also found after multiple sessions of stimulation (Hedges’ g = 0.81, 95% CI = 0.51—1.11, p < 0.001, I^2^ = 41.51%), although the publication bias was significant (p < 0.001). It was noted that the increased MEP amplitude could be sustained for almost three months [62, 63, 68].

Conversely, the MEP amplitude of the unaffected M1 decreased by 21.29% (95% CI = − 27.17% to − 15.41%, p < 0.001, I^2^ = 56.88%) immediately after a single session of LF-rTMS, in line with two other studies [41, 42]; however, the publication bias was significant (p = 0.040). Likewise, the MEP amplitude of the unaffected M1 decreased after multiple sessions of LF-rTMS (Hedges’ g = − 0.54, 95% CI = − 0.78 to − 0.30, p < 0.001, I^2^ = 26.05%), with no significant publication bias identified (p = 0.502). In terms of its long-term effects, the decreased MEP amplitude could be sustained for three to six months after the intervention [59, 62, 63].

#### Intracortical inhibition/facilitation

Despite the heterogeneity of the inter-stimulus intervals and intensities, four studies consistently found no significant changes in regard to the SICI [24, 26, 32, 36], cortical silent period [32, 36], and ICF [32] of the affected M1 after a single session of LF-rTMS. Similarly, no significant changes were found in the unaffected M1 [36, 55, 60]. Only one study conducted by Mello et al. revealed that the ICF of the unaffected M1 was upregulated after 10 sessions of LF-rTMS in patients with stroke at the acute/subacute stage [60].

#### Transcallosal inhibition

A limited number of studies have suggested that iSP recorded in the affected hand reduced immediately after a single session [9, 28] and 10 sessions [50] of LF-rTMS. For the unaffected hand, no significant effects on iSP were found [28, 32]. By measuring IHI, decreased inhibition from the unaffected to the affected M1 was also noted [32].

### High frequency rTMS to the affected M1

Seven [10, 26, 29, 30, 33, 35, 41] and 13 [43, 45, 47, 48, 61–63, 66, 70, 73, 74, 76, 77] studies evaluated the effects of a single session and multiple sessions of HF-rTMS on the affected M1 in cortical excitability, respectively. Khedr et al. showed that more patients presented MEP responses after receiving 10 sessions of real HF-rTMS than sham [43]. However, this comparison did not yield statistical significance.

#### Resting/active motor threshold

**Fig. 3:**
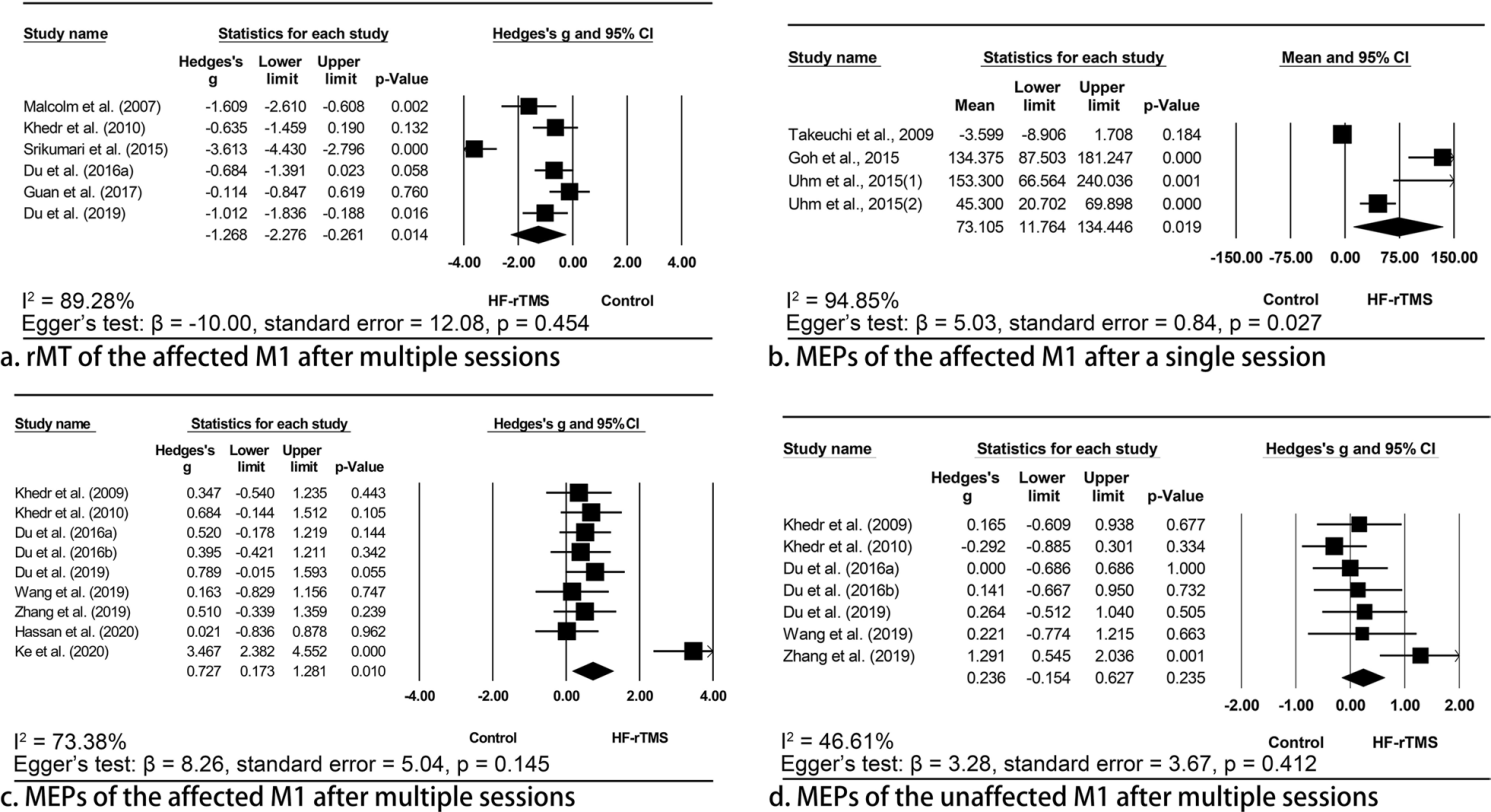
Meta-analyses indicating the effects of high frequency repetitive transcranial magnetic stimulation (HF-rTMS) to the affected M1 in modulating bilateral cortical excitability. A meta-analysis indicated that multiple sessions of HF-rTMS applied to the affected M1 significantly decreased the rMT of the affected M1 (**a**). A single session of HF-rTMS significantly increases MEP amplitudes of the affected M1 by 73.11% (**b**). Multiple sessions of HF-rTMS also significantly increased MEP amplitudes of the affected M1 (**c**). However, multiple sessions of stimulation had no effects on the MEP amplitude of the unaffected M1 (**d**). *rMT* resting motor threshold, *MEP* motor-evoked potentials

A single session of HF-rTMS was shown to decrease the rMT [29] and aMT [47, 48] of the affected M1; a meta-analysis also supported the notion that multiple sessions of HF-rTMS could decrease the rMT of the affected M1 (Hedges’ g = − 1.27, 95% CI = − 2.28 to − 0.26, p = 0.014, I^2^ = 89.28%; Fig. 3) without evident publication bias (p = 0.454). The effects on the unaffected M1 were less investigated and inconsistent results were reported [47, 48, 62, 70].

#### Amplitude of motor-evoked potentials

A single session of HF-rTMS did not show any effects on the MEP amplitude of the unaffected M1 [26, 41], while it significantly increased that of the affected M1 [10, 41]. A meta-analysis showed an MEP increment of 73.11% (95% CI = 11.76%–134.45%, p = 0.019, I^2^ = 94.85%); however, significant publication bias was identified (p = 0.027). Likewise, meta-analyses of multiple-session studies also supported the notion that HF-rTMS had no effects on the MEP amplitude of the unaffected M1 (Hedges’ g = 0.24, 95% CI = − 0.15–0.63, p = 0.235, I^2^ = 46.61%), but significantly increased that of the affected M1 (Hedges’ g = 0.73, 95% CI = 0.17 –1.28, p = 0.010, I^2^ = 73.38%). No publication bias was identified in the former (p = 0.412) and latter (p = 0.145) analyses.

#### Intracortical inhibition/facilitation

It was consistently found that a single session of HF-rTMS did not change the SICI or the ICF of the affected M1 [26, 29, 30].

### Intermittent theta burst stimulation to the affected M1

The effects of a single session of iTBS were extensively studied [14–17, 27, 37–40], and four studies investigated the effects of multiple sessions of iTBS on cortical excitability [53, 54, 57, 64, 68]. iTBS was given at subthreshold intensities, and almost all the studies delivered 600 pulses, except the study by Hsu et al. in which patients received 1200 pulses [53].

#### Resting/active motor threshold

**Fig. 4:**
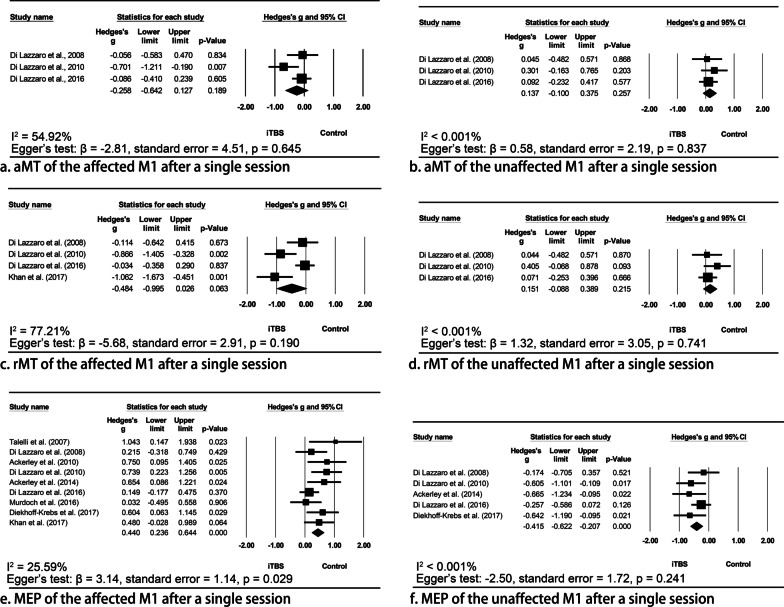
Meta-analyses indicating the effects of intermittent theta burst stimulation (iTBS) to the affected M1 in modulating bilateral cortical excitability. Meta-analyses indicated that a single session of iTBS tended to decrease the aMT (**a**) and rMT (**c**) of the affected M1 and increase the aMT (**b**) and rMT (**d**) of the unaffected M1. However, none of the pooled Hedges’ g values were significant. A single session of iTBS significantly increased the MEP amplitude of the affected M1 (**e**) and decreased that of the unaffected M1 (**f**). *rMT* resting motor threshold; *aMT* active motor threshold; *MEP* motor-evoked potentials

Meta-analyses indicated that a single session of iTBS tended to decrease the aMT (Hedges’ g = − 0.26, 95% CI = − 0.64 to 0.13, p = 0.189, I^2^ = 54.92%) and rMT (Hedges’ g = − 0.48, 95% CI =− 1.00 to 0.03, p = 0.063, I^2^ = 77.21%) of the affected M1, and increase the aMT (Hedges’ g = 0.14, 95% CI = − 0.10 to 0.38, p = 0.257, I2 < 0.001%) and rMT (Hedges’ g = 0.15, 95% CI = − 0.09 to 0.39, p = 0.215, I^2^ < 0.001%) of the unaffected M1; however, none of the pooled Hedges’ g values was significant (Fig. 4). No publication bias was identified in any of the meta-analyses (all p values > 0.05).

For the effects of multiple sessions of stimulation, iTBS combined with physical therapy was beneficial to the rMT and aMT of the bilateral M1s [53, 54, 57, 64]. It is noteworthy that the post-iTBS measurements were conducted one day after the last session of the intervention in two studies [53, 64], rather than immediately after the stimulation in those studies investigating the effects of a single session of iTBS.

#### Amplitude of motor evoked potentials

A single session of iTBS significantly increased the MEP amplitude of the affected M1 (Hedges’ g = 0.44, 95% CI = 0.24 to 0.64, p < 0.001, I^2^ = 25.59%) and decreased that of the unaffected M1 (Hedges’ g = − 0.42, 95% CI = − 0.62 to − 0.21, p < 0.001, I^2^ < 0.001%). Significant publication bias was identified in the former analysis (p = 0.029), but not in the latter one (p = 0.241).

Largely inconsistent findings were reported regarding the MEP amplitudes of the bilateral M1s after multiple sessions of iTBS [53, 54, 57, 64]. For the long-term effects, a previous study revealed that iTBS had superior effects in sustaining the increased cortical excitability in the affected M1 for almost three months [68].

#### Intracortical inhibition

Preliminary evidence did not support the modulatory effects of iTBS on SICI [38] and the cortical silent period of the affected M1 in patients with stroke [40].

### Continuous theta burst stimulation to the unaffected M1

A study conducted by Di Lazzaro et al. suggested that a single session of cTBS to the unaffected M1 had similar effects as iTBS to the affected M1, simultaneously increasing the rMT and aMT of the unaffected M1 and decreasing those of the affected M1 [15]. However, another study, also conducted by Di Lazzaro et al. showed that 10 sessions of cTBS did not effectively modulate cortical excitability, reflected by the nonsignificant changes in the rMT, aMT, and MEP amplitudes of the bilateral M1s [52]. These nonsignificant findings were also confirmed by Neva et al. in which neither the SICI, the ICF, nor the iSP of the bilateral M1s benefited from cTBS [72].

### Correlation between the changes in TMS measures and motor improvement

A total of 12 studies performed various correlation analyses between TMS measures and motor impairment [43, 48–50, 56, 57, 59, 61–63, 70, 71, 75]. Cross-sectional analyses indicated positive correlations between the cortical excitability of the affected M1 and the severity of motor impairment of the hemiplegic arm [61, 63], while that of the unaffected M1 was negatively correlated with motor impairment of the hemiplegic arm [71]. Furthermore, motor improvement was significantly correlated with a reduction in the cortical excitability of the unaffected M1 [56, 57, 59]. The decrease in iSP from the unaffected to the affected M1 and the increase in MEP amplitudes in the affected M1 were correlated with arm motor improvement [50, 62]. A previous study has also shown the predictive value of MEP amplitudes for subsequent motor improvement [48].

Two studies conducted these analyses in patients with severe arm impairments. Positive correlation between the cortical excitability of the unaffected M1 and arm motor impairment was noted after receiving LF-rTMS [49]. The changes in MEP latency in the unaffected M1 were negatively correlated with motor improvement [75].

## Discussion

The aim of the current study was to systematically review the effects of four commonly used rTMS protocols on the cortical excitability of patients with a unilateral stroke lesion. LF-rTMS to the unaffected M1 is the most extensively investigated one in the literature. Our meta-analyses indicated that it was effective in increasing the MEP amplitude and decreasing the rMT of the affected M1, with opposite effects regarding the unaffected M1. Applying HF-rTMS to the affected M1 increased cortical excitability of the stimulated M1, while no significant effects were found on that of the unaffected M1. Unlike the modulatory effects of HF-rTMS, iTBS not only increased the cortical excitability of the affected M1, but also decreased that of the unaffected M1. A limited number of studies investigated cTBS in stroke and a firm conclusion cannot be drawn. Motor impairment of the hemiplegic arm was significantly correlated with various forms of TMS measures.

The meta-analysis conducted by McDonnell et al. suggested that the unaffected M1 of patients with stroke was not hyperactivated during active contraction and resting, indicated by the nonsignificant differences on aMT, rMT, and MEPs compared with healthy controls [1]. Most recently, a longitudinal study revealed that the premovement IHI did not significantly differ from that of healthy controls at the acute/subacute stage of stroke, however, the premovement IHI was abnormally higher at the chronic stage [79]. Therefore, the authors argued that the excessive IHI in chronic stroke patients may be a consequence of cortical reorganization, but it was not causally associated with motor impairment [79]. To the best of our knowledge, this study suggested that motor impairment at the acute/subacute stage was not caused by excessive premovement. However, the study by Xu et al. [79] cannot rule out the possibility that the interhemispheric imbalance developed at the chronic stage, presumably caused by the maladaptation of cortical reorganization or learned non-use, may prevent the recovery of motor function, since many studies have supported the clinical effects of LF-rTMS to the unaffected M1 in chronic stroke patients. In our meta-analyses, we found that LF-rTMS to the unaffected M1 could significantly increase the cortical excitability of the affected M1, accompanied by decreased cortical excitability of the unaffected M1. A small number of studies also documented that reduced iSP and IHI after LF-rTMS were highly correlated with motor improvement [9, 28, 50], which would further reinforce the interhemispheric imbalance model.

In a large-scale randomized controlled trial by Harvey et al. [80], the authors failed to find superior effects of LF-rTMS applied to the unaffected M1 against sham stimulation in improving poststroke upper limb motor functions. The failure to reject the null hypothesis points out the importance of studying the characteristics of treatment responders to LF-rTMS. Carey et al. found that the responders to LF-rTMS (applied to the unaffected M1) had greater hand function and greater preservation volume of the ipsilesional posterior limb of the internal capsule at baseline, compared with the nonresponders [81]. Besides, disrupting either the unaffected M1 or the unaffected dorsal pre-motor cortex by TMS worsened the contralateral motor performance in stroke patients with severe motor impairment [82], and that HF-rTMS applied to the unaffected M1 could facilitate motor improvement in stroke patients with severe motor impairment [75]. These studies suggested that the unaffected hemisphere plays different roles in motor recovery according to the severity of motor impairment [75, 81, 82]. Di Pino et al. proposed a bimodal balance–recovery model to explain the differential roles of the unaffected hemisphere in poststroke motor recovery [8]. Patients with mild motor impairment can benefit from inhibitory brain stimulation applied to the unaffected hemisphere due to high level of structural preservation, whereas the unaffected hemisphere, because of substantial damage to the ipsilesional corticospinal tract, has a significant role for compensation. Recently, Lin et al. found that IHI from the unaffected to affected M1 was negatively correlated with upper limb function in less impaired patients, while positive correlation was found in more impaired patients [83]. This supports further the bimodal balance–recovery model. In our current systematic review, only one study in which HF-rTMS was applied to the unaffected M1 in patients with severe motor impairment, and this study showed that improvement in upper limb function was correlated with shortened latency of unaffected MEP [75]. Because MEPs from the affected M1 was not detectable [75], it remained unknown whether the excitability of the affected M1 was altered after HF-rTMS applied to the unaffected M1. Regarding the mechanism, recent studies are still debating the pathway of the unaffected M1 in facilitating motor recovery via callosal connections, or direct pathway from the unaffected hemisphere [84, 85]. Traditional measures of intracortical and interhemispheric excitability/inhibition rely highly on electromyography outputs, which are usually not detectable in patients with severe motor impairment. Alternatively, combining TMS and electroencephalography can directly probe the cortical reactivity after TMS pulses [86, 87], which might be useful in the investigation of interhemispheric communication for those patients with severe motor impairments.

As aforementioned, the neurophysiological effects of LF-rTMS to the unaffected M1 can be explained by the mechanism of rebalancing interhemispheric inhibition. It is necessary to understand whether hyperactivity or excessive inhibition flow from the unaffected M1 is a prerequisite for LF-rTMS applied to the unaffected M1 aiming to modulate the cortical excitability of the affected M1. Healthy people are assumed to have balanced interhemispheric inhibition. However, many previous studies have found that LF-rTMS and cTBS not only suppressed the MEP amplitude of the simulated M1, but also enhanced that of the non-stimulated M1 [88, 89]. Increased cortical excitability within the non-stimulated M1 is likely relevant to the elevated intrinsic excitability of the excitatory interneurons responsible for glutamatergic non-NMDA receptors [90]. Given the above findings in healthy people, it is reasonable to expect the cortical excitability of the affected M1 to benefit from inhibitory stimulation to the unaffected M1, even though the unaffected M1 is not hyperactivated or does not inhibit the affected M1 excessively during resting and premovement at the acute and subacute stages after stroke [1, 79]. If this is the case, the interhemispheric imbalance model may not be the unique hypothesis of inhibitory stimulation to the unaffected M1, and future studies are warranted.

A large body of evidence suggests that the cortical excitability of the affected M1 decreased after stroke, and motor improvement is associated with the increase of excitability in the affected M1 [1]. Our meta-analyses indicated that both HF-rTMS and iTBS are useful in increasing the cortical excitability of the unaffected M1. Furthermore, the effect of simultaneously suppressing the unaffected M1 was noted after iTBS, but not after HF-rTMS. For a direct comparison, a previous study has shown the superior effects of iTBS in increasing the MEPs of the stimulated M1, compared to HF-rTMS in healthy people [88], but comparable effects were also reported [91]. Animal studies suggested that different TMS protocols may have specifically different effects in modulating neurogenesis and protein expression, which may potentially account for the different effects across neurophysiological and clinical measures [92].

In addition to unilateral-hemispheric stimulation, some new protocols integrating these regular forms of rTMS have been studied in stroke. For instance, the dual-hemisphere stimulation consisting of inhibitory rTMS to the unaffected M1 and excitatory rTMS to the affected M1 was more effective in enhancing motor performance and cortical excitability [74] and reducing the SICI of the affected M1 than unilateral-hemispheric stimulation [26]. Another protocol, LF-rTMS primed with HF-rTMS, also showed encouraging effects in reducing intracortical inhibition within the affected M1 [32]. Besides, functional rTMS triggered by electromyogram may induce greater excitability changes than passive stimulation protocols [29].

### Limitations

This review was not free from limitations. First, we must be cautious when interpreting the findings relevant to LF-rTMS. The high IHI elicited by paired-pulse TMS was found during the premovement period but the majority of previous TMS measures were conducted at rest. Therefore, the outcomes—paired-pulse induced IHI at rest and single-pulse induced iSP during sustained isometric contractions, in our review might not be conclusive in explaining excessive inhibition driving from the unaffected M1 [89]. Second, substantial heterogeneity and publication bias were identified in some meta-analyses, probably due to the small sample sizes in most included studies and the way in which the patient characteristics, TMS protocols, and methodologies were not identical. Third, the available studies mostly focused on the effects on TMS measures, which may limit our findings to patients with mild to moderate impairments only. This is because the MEPs of the affected M1 are usually not recordable in stroke patients with severe motor impairments.

## Conclusions

The current study systematically reviewed existing research investigating the effects of four forms of rTMS in modulating the cortical excitability of bilateral M1s. LF-rTMS to the unaffected M1 is the most extensively studied protocol, while cTBS is the least studied one. Although recent studies have argued for the rationale of inhibitory stimulation applied to the unaffected M1, our analyses suggested that LF-rTMS not only suppressed the cortical excitability of the unaffected M1 but also simultaneously enhanced that of the affected M1. HF-rTMS enhanced the cortical excitability of the affected M1 only. Preliminary evidence also supported the effects of iTBS in rebalancing bilateral cortical excitability in stroke. Our findings support the bimodal balance–recovery model in patients with mild motor impairment, more studies are needed to investigate the neurophysiological effects of HF-rTMS applied to the unaffected M1 in patients with severe motor impairment.

## Supplementary Information

**Additional file 1: Table S1** The methodological quality of transcranial magnetic stimulation studies investigating the effects of a single session of simulation on cortical excitability. **Table. S2** The methodological quality of transcranial magnetic stimulation studies investigating the effects of multiple sessions of simulation on cortical excitability. **Figs. S1 to S17** The funnel plots for the meta-analysis regarding the effects of rTMS on various outcomes.

## Data Availability

All data generated or analyzed during this study are included in this published article and its additional file.

## Abbreviations

TMS: Transcranial magnetic stimulation
M1: Primary motor cortex
MEPs: Motor evoked potentials
rMT: Resting motor threshold
aMT: Active motor threshold
rTMS: Repetitive transcranial magnetic stimulation
SICI: Short-interval intracortical inhibition
IHI: Interhemispheric inhibition
iSP: Ipsilateral silent period
LF-rTMS: Low frequency rTMS
HF-rTMS: High frequency rTMS
TBS: Theta burst stimulation
iTBS: Intermittent theta burst stimulation
cTBS: Continuous theta burst stimulation
ICF: Intracortical facilitation
