# Supplementary material for: Effects of transcranial magnetic stimulation in modulating cortical excitability in patients with stroke: a systematic review and meta-analysis
Source: J Neuroeng Rehabil. 2022 Feb 22;19:24. doi: 10.1186/s12984-022-00999-4 (PMC8862292; doi:10.1186/s12984-022-00999-4)
Supplement: Supplementary file 1 — Additional file 1: Table S1 The methodological quality of transcranial magnetic stimulation studies investigating the effects of a single session of simulation on cortical excitability. Table. S2 The methodological quality of transcranial magnetic stimulation studies investigating the effects of multiple sessions of simulation on cortical excitability. Figs. S1 to S17 The funnel plots for the meta-analysis regarding the effects of rTMS on various outcomes. [file 12984_2022_999_MOESM1_ESM.docx]

Supplementary material

Table S1 The methodological quality of transcranial magnetic stimulation studies investigating the effects of a single session of simulation on cortical excitability

|  | Participant factors | | | | | | | Methodological factors | | | | | | | | | | | | | | | | | | | | Analytical factors | |
| --- | --- | --- | --- | --- | --- | --- | --- | --- | --- | --- | --- | --- | --- | --- | --- | --- | --- | --- | --- | --- | --- | --- | --- | --- | --- | --- | --- | --- | --- |
|  |  |  |  |  |  |  |  |  |  |  |  |  |  |  |  |  |  |  |  |  |  |  |  |  |  |  |  |  |  |
| Study | Age | Gender | Handedness | Medication | CNS drugs | Medical condition | History of motor activity | EMG electrodes | Relaxation/contraction of target muscles | Motor activity before testing | Relaxation of muscles other than those being tested | Coil type | Coil orientation | Direction of induced current | Coil location and stability | Type of stimulator | Stimulation intensity | Pulse shape | Optimal hotspot | Time between MEPs | Time between testing | Subject attention | Threshold | Number of MEPs | Paired pulse only | | | MEP size during analysis | Size of unconditioned MEP |
|  |  |  |  |  |  |  |  |  |  |  |  |  |  |  |  |  |  |  |  |  |  |  |  |  | Test pulse | Conditioning pulse | Inter-stimulus interval |  |  |
| Takeuchi et al (2005) | 1 | 1 | 0 | 0 | 0 | 1 | 0 | 1 | 1 | 1 | 1 | 1 | 0 | 0 | 0 | 1 | 1 | 0 | 1 | 1 | N/A | 0 | 1 | 1 | N/A | N/A | N/A | 1 | 1 |
| Kim et al (2006) | 1 | 1 | 0 | 1 | 0 | 1 | 0 | 1 | 1 | 1 | 1 | 1 | 1 | 0 | 0 | 1 | 1 | 0 | 1 | 1 | 1 | 0 | 1 | 1 | N/A | N/A | N/A | 1 | 1 |
| Talelli et al (2007) | 1 | 1 | 0 | 0 | 0 | 1 | 0 | 1 | 1 | 1 | 1 | 1 | 1 | 0 | 0 | 1 | 1 | 1 | 1 | 0 | 1 | 1 | 1 | 1 | N/A | N/A | N/A | 1 | 1 |
| Di Lazzaro et al (2008) | 1 | 1 | 0 | 0 | 1 | 1 | 0 | 1 | 0 | 0 | 1 | 1 | 1 | 1 | 0 | 1 | 1 | 0 | 1 | 0 | 1 | 0 | 1 | 1 | N/A | N/A | N/A | 1 | 1 |
| Takeuchi et al (2008) | 1 | 1 | 0 | 0 | 0 | 1 | 0 | 1 | 1 | 0 | 1 | 1 | 1 | 0 | 0 | 1 | 1 | 0 | 1 | 0 | 0 | 0 | 1 | 1 | 1 | 1 | 1 | 1 | 1 |
| Jayaram et al (2009) | 1 | 1 | 0 | 0 | 0 | 1 | 0 | 1 | 1 | 0 | 1 | 1 | 1 | 1 | 0 | 1 | 1 | 1 | 1 | 1 | 1 | 0 | 1 | 1 | N/A | N/A | N/A | 1 | 1 |
| Takeuchi et al (2009) | 1 | 1 | 0 | 0 | 0 | 1 | 0 | 1 | 1 | 0 | 1 | 1 | 0 | 0 | 0 | 1 | 1 | 0 | 1 | 0 | N/A | 0 | 1 | 1 | 1 | 1 | 1 | 1 | 1 |
| Ackerley et al (2010) | 1 | 1 | 0 | 0 | 0 | 1 | 0 | 1 | 1 | 1 | 1 | 1 | 0 | 0 | 0 | 1 | 1 | 1 | 0 | 0 | 1 | 0 | 0 | 1 | N/A | N/A | N/A | 1 | 1 |
| Di Lazzaro et al (2010) | 1 | 1 | 0 | 0 | 1 | 1 | 0 | 1 | 0 | 1 | 1 | 1 | 1 | 1 | 0 | 1 | 1 | 0 | 1 | 0 | N/A | 0 | 1 | 1 | 1 | 1 | 1 | 1 | 1 |
| Takeuchi et al (2012) | 1 | 1 | 0 | 0 | 1 | 1 | 0 | 0 | 0 | 1 | 1 | 1 | 0 | 0 | 0 | 1 | 1 | 0 | 1 | 0 | 0 | 0 | 1 | 1 | N/A | N/A | N/A | 1 | 1 |
| Massie et al (2013) | 1 | 1 | 0 | 0 | 1 | 1 | 0 | 1 | 1 | 1 | 1 | 1 | 1 | 1 | 0 | 1 | 1 | 0 | 1 | 0 | N/A | 0 | 1 | 0 | 1 | 1 | 1 | 1 | 1 |
| Massie et al (2013) | 1 | 1 | 0 | 0 | 0 | 1 | 0 | 1 | 1 | 1 | 1 | 1 | 1 | 0 | 0 | 1 | 1 | 0 | 1 | 0 | N/A | 0 | 1 | 0 | 1 | 1 | 1 | 1 | 1 |
| Ackerley et al (2014) | 1 | 1 | 0 | 0 | 1 | 1 | 0 | 1 | 1 | 1 | 1 | 1 | 0 | 0 | 0 | 1 | 1 | 0 | 1 | 0 | 1 | 0 | 1 | 1 | N/A | N/A | N/A | 1 | 1 |
| Vongvaivanichakul et al (2014) | 1 | 0 | 0 | 0 | 0 | 1 | 0 | 1 | 1 | 1 | 1 | 1 | 0 | 0 | 0 | 1 | 1 | 0 | 1 | 0 | N/A | 0 | 0 | 0 | N/A | N/A | N/A | 1 | 1 |
| Cassidy et al (2015) | 1 | 1 | 1 | 0 | 1 | 1 | 0 | 1 | 1 | 1 | 1 | 1 | 1 | 0 | 0 | 1 | 1 | 0 | 1 | 0 | 1 | 0 | 1 | 1 | 1 | 1 | 1 | 1 | 1 |
| Goh et al (2015) | 1 | 1 | 0 | 0 | 0 | 1 | 0 | 1 | 1 | 1 | 1 | 1 | 0 | 0 | 0 | 1 | 1 | 0 | 1 | 0 | 1 | 0 | 1 | 0 | N/A | N/A | N/A | 1 | 1 |
| Tretriluxana et al (2015) | 1 | 1 | 1 | 0 | 0 | 0 | 0 | 1 | 1 | 1 | 1 | 1 | 0 | 0 | 0 | 1 | 1 | 0 | 1 | 0 | 1 | 0 | 1 | 1 | N/A | N/A | N/A | 1 | 1 |
| Uhm et al (2015) | 1 | 1 | 0 | 0 | 0 | 1 | 0 | 1 | 1 | 0 | 1 | 1 | 1 | 0 | 0 | 1 | 1 | 0 | 1 | 0 | 1 | 0 | 1 | 1 | N/A | N/A | N/A | 1 | 1 |
| Bashir et al (2016) | 1 | 1 | 0 | 0 | 0 | 0 | 0 | 1 | 1 | 0 | 1 | 1 | 0 | 0 | 1 | 1 | 1 | 1 | 1 | 1 | N/A | 1 | 1 | 1 | N/A | N/A | N/A | 1 | 1 |
| Di Lazzaro et al (2016) | 1 | 1 | 0 | 0 | 1 | 1 | 0 | 1 | 0 | 0 | 1 | 1 | 1 | 1 | 1 | 1 | 1 | 0 | 1 | 0 | N/A | 0 | 1 | 0 | N/A | N/A | N/A | 1 | 1 |
| Murdoch et al (2016) | 1 | 1 | 0 | 0 | 1 | 1 | 0 | 1 | 1 | 1 | 1 | 1 | 1 | 0 | 1 | 1 | 1 | 0 | 1 | 1 | 1 | 0 | 1 | 1 | 1 | 1 | 1 | 1 | 1 |
| Diekhoff-Krebs et al (2017) | 1 | 1 | 1 | 0 | 0 | 1 | 0 | 1 | 1 | 1 | 1 | 1 | 1 | 0 | 1 | 1 | 1 | 0 | 1 | 1 | 1 | 1 | 1 | 1 | N/A | N/A | N/A | 1 | 1 |
| Khan et al (2017) | 1 | 1 | 0 | 0 | 0 | 1 | 0 | 1 | 1 | 1 | 1 | 1 | 1 | 1 | 1 | 1 | 1 | 0 | 1 | 0 | N/A | 0 | 1 | 1 | N/A | N/A | N/A | 1 | 1 |
| Hanafi et al (2018) | 1 | 1 | 0 | 0 | 0 | 1 | 0 | 0 | 1 | 1 | 1 | 1 | 0 | 0 | 0 | 1 | 0 | 0 | 1 | 0 | 1 | 0 | 1 | 0 | N/A | N/A | N/A | 1 | 1 |
| Tretriluxana et al (2018) | 1 | 1 | 0 | 0 | 0 | 1 | 0 | 1 | 1 | 1 | 1 | 1 | 0 | 0 | 0 | 1 | 1 | 0 | 1 | 0 | N/A | 0 | 1 | 0 | N/A | N/A | N/A | 1 | 1 |
| Note: 1 = reported. 0 = not reported. | | | | | | | | | | | | | | | | | | | | | | | | | | | | | |

Table S2 The methodological quality of transcranial magnetic stimulation studies investigating the effects of multiple sessions of simulation on cortical excitability

|  | Participant factors | | | | | | | Methodological factors | | | | | | | | | | | | | | | | | | | | Analytical factors | |
| --- | --- | --- | --- | --- | --- | --- | --- | --- | --- | --- | --- | --- | --- | --- | --- | --- | --- | --- | --- | --- | --- | --- | --- | --- | --- | --- | --- | --- | --- |
|  |  |  |  |  |  |  |  |  |  |  |  |  |  |  |  |  |  |  |  |  |  |  |  |  |  |  |  |  |  |
| Studies | Age | Gender | Handedness | Medication | CNS drugs | Medical condition | History of motor activity | EMG electrodes | Relaxation/contraction of target muscles | Motor activity before testing | Relaxation of muscles other than those being tested | Coil type | Coil orientation | Direction of induced current | Coil location and stability | Type of stimulator | Stimulation intensity | Pulse shape | Optimal hotspot | Time between MEPs | Time between testing | Subject attention | Threshold | Number of MEPs | Paired pulse only | | | MEP size during analysis | Size of unconditioned MEP |
|  |  |  |  |  |  |  |  |  |  |  |  |  |  |  |  |  |  |  |  |  |  |  |  |  | Test pulse | Conditioning pulse | Inter-stimulus interval |  |  |
| Khedr et al (2005) | 1 | 1 | 1 | 0 | 1 | 1 | 1 | 0 | 1 | 1 | 1 | 1 | 0 | 0 | 0 | 1 | 0 | 0 | 1 | 0 | 1 | 0 | 1 | 0 | N/A | N/A | N/A | 1 | 1 |
| Fregni et al (2006) | 1 | 1 | 0 | 1 | 1 | 1 | 1 | 0 | 1 | 0 | 1 | 0 | 0 | 0 | 0 | 0 | 0 | 0 | 1 | 0 | 1 | 0 | 0 | 0 | N/A | N/A | N/A | 1 | 1 |
| Malcolm et al (2007) | 1 | 1 | 1 | 1 | 1 | 1 | 1 | 1 | 1 | 1 | 1 | 1 | 0 | 0 | 0 | 1 | 1 | 0 | 1 | 0 | 1 | 0 | 1 | 0 | N/A | N/A | N/A | 1 | 1 |
| Pomeroy et al (2007) | 1 | 1 | 0 | 0 | 0 | 1 | 1 | 1 | 1 | 1 | 1 | 1 | 1 | 0 | 0 | 1 | 1 | 0 | 1 | 1 | 1 | 0 | 1 | 0 | N/A | N/A | N/A | 0 | 0 |
| Khedr et al (2009) | 1 | 1 | 0 | 0 | 0 | 1 | 1 | 1 | 0 | 1 | 1 | 1 | 1 | 0 | 0 | 1 | 1 | 0 | 1 | 1 | 1 | 0 | 1 | 1 | N/A | N/A | N/A | 1 | 1 |
| Khedr et al (2010) | 1 | 1 | 0 | 0 | 0 | 1 | 1 | 1 | 0 | 1 | 1 | 1 | 1 | 0 | 0 | 1 | 1 | 0 | 1 | 1 | 1 | 0 | 1 | 1 | N/A | N/A | N/A | 1 | 1 |
| Theilig et al (2011) | 1 | 1 | 0 | 0 | 0 | 1 | 1 | 1 | 1 | 1 | 1 | 1 | 1 | 0 | 1 | 1 | 1 | 0 | 1 | 0 | 1 | 0 | 1 | 1 | N/A | N/A | N/A | 1 | 1 |
| Avenanti et al (2012) | 1 | 1 | 0 | 0 | 0 | 1 | 1 | 1 | 1 | 1 | 1 | 1 | 1 | 0 | 0 | 1 | 1 | 0 | 1 | 0 | 1 | 0 | 1 | 0 | N/A | N/A | N/A | 1 | 1 |
| Wang et al (2012) | 1 | 1 | 0 | 0 | 0 | 1 | 1 | 1 | 1 | 1 | 1 | 1 | 1 | 1 | 0 | 1 | 1 | 0 | 1 | 1 | 1 | 0 | 1 | 1 | N/A | N/A | N/A | 1 | 1 |
| Di Lazzaro et al (2013) | 1 | 1 | 0 | 1 | 0 | 1 | 1 | 1 | 0 | 0 | 1 | 1 | 1 | 1 | 0 | 1 | 1 | 0 | 1 | 0 | 1 | 0 | 1 | 0 | N/A | N/A | N/A | 1 | 1 |
| Hsu et al (2013) | 1 | 1 | 0 | 0 | 0 | 1 | 1 | 1 | 1 | 1 | 1 | 1 | 1 | 1 | 0 | 1 | 1 | 0 | 1 | 0 | 1 | 0 | 1 | 1 | N/A | N/A | N/A | 1 | 1 |
| Sung et al (2013) | 1 | 1 | 0 | 0 | 0 | 1 | 1 | 1 | 1 | 1 | 1 | 1 | 0 | 0 | 0 | 1 | 1 | 0 | 1 | 1 | 1 | 0 | 1 | 1 | N/A | N/A | N/A | 1 | 1 |
| Rose et al (2014) | 1 | 1 | 0 | 0 | 0 | 1 | 1 | 1 | 1 | 1 | 1 | 1 | 1 | 0 | 0 | 1 | 1 | 0 | 1 | 1 | 1 | 0 | 1 | 1 | 1 | 1 | 1 | 1 | 1 |
| Wang et al (2014a) | 1 | 1 | 0 | 0 | 0 | 1 | 1 | 1 | 1 | 1 | 1 | 1 | 1 | 1 | 0 | 1 | 1 | 0 | 1 | 1 | 1 | 0 | 1 | 1 | N/A | N/A | N/A | 1 | 1 |
| Wang et al (2014b) | 1 | 1 | 0 | 0 | 0 | 1 | 1 | 1 | 1 | 1 | 1 | 1 | 1 | 0 | 0 | 1 | 1 | 0 | 1 | 1 | 1 | 0 | 1 | 1 | N/A | N/A | N/A | 1 | 1 |
| Blesneag et al (2015) | 1 | 1 | 0 | 0 | 0 | 1 | 1 | 1 | 1 | 1 | 1 | 1 | 1 | 0 | 0 | 1 | 1 | 0 | 1 | 1 | 1 | 0 | 1 | 1 | N/A | N/A | N/A | 1 | 1 |
| Ludemann-Podubecka et al (2015) | 1 | 1 | 1 | 0 | 0 | 1 | 1 | 1 | 1 | 1 | 1 | 1 | 1 | 1 | 0 | 1 | 1 | 0 | 1 | 0 | 1 | 0 | 1 | 1 | N/A | N/A | N/A | 1 | 1 |
| Mello et al (2015) | 1 | 1 | 1 | 0 | 0 | 1 | 1 | 1 | 1 | 1 | 1 | 1 | 1 | 0 | 0 | 1 | 1 | 1 | 1 | 0 | 1 | 0 | 1 | 0 | 1 | 1 | 1 | 1 | 1 |
| Srikumari et al (2015) | 1 | 0 | 0 | 0 | 0 | 1 | 1 | 0 | 1 | 1 | 1 | 1 | 1 | 0 | 0 | 1 | 1 | 0 | 0 | 0 | 1 | 0 | 0 | 0 | N/A | N/A | N/A | 1 | 1 |
| Du et al (2016a) | 1 | 1 | 0 | 0 | 0 | 1 | 1 | 1 | 1 | 1 | 1 | 1 | 1 | 0 | 0 | 1 | 1 | 0 | 0 | 0 | 1 | 0 | 1 | 0 | N/A | N/A | N/A | 1 | 1 |
| Du et al (2016b) | 1 | 1 | 0 | 0 | 0 | 1 | 1 | 1 | 1 | 1 | 1 | 1 | 0 | 0 | 0 | 1 | 1 | 0 | 1 | 0 | 1 | 0 | 1 | 0 | N/A | N/A | N/A | 1 | 1 |
| Volz et al (2016) | 1 | 1 | 1 | 0 | 0 | 1 | 1 | 1 | 1 | 1 | 1 | 1 | 1 | 1 | 1 | 1 | 1 | 1 | 1 | 1 | 1 | 0 | 1 | 1 | N/A | N/A | N/A | 1 | 1 |
| Cha et al (2017) | 1 | 1 | 0 | 0 | 1 | 1 | 1 | 1 | 1 | 1 | 1 | 1 | 1 | 0 | 1 | 1 | 1 | 0 | 1 | 1 | 1 | 0 | 1 | 1 | N/A | N/A | N/A | 1 | 1 |
| Guan et al (2017) | 1 | 1 | 0 | 0 | 0 | 1 | 1 | 0 | 1 | 1 | 1 | 1 | 1 | 0 | 1 | 1 | 1 | 0 | 0 | 0 | 1 | 0 | 1 | 0 | N/A | N/A | N/A | 1 | 1 |
| Huang et al (2018) | 1 | 1 | 0 | 0 | 0 | 1 | 1 | 1 | 1 | 1 | 1 | 1 | 0 | 0 | 0 | 1 | 1 | 0 | 1 | 1 | 1 | 0 | 1 | 1 | N/A | N/A | N/A | 1 | 1 |
| Watanabe et al (2018) | 1 | 1 | 0 | 0 | 1 | 1 | 1 | 0 | 1 | 1 | 1 | 1 | 0 | 0 | 0 | 1 | 1 | 0 | 0 | 0 | 1 | 0 | 0 | 1 | N/A | N/A | N/A | 1 | 1 |
| Dos Santos et al (2019) | 1 | 1 | 0 | 0 | 0 | 1 | 1 | 1 | 1 | 1 | 1 | 1 | 1 | 0 | 0 | 1 | 1 | 0 | 1 | 0 | 1 | 0 | 1 | 0 | N/A | N/A | N/A | 1 | 1 |
| Du et al (2019) | 1 | 1 | 0 | 0 | 1 | 1 | 1 | 0 | 1 | 1 | 1 | 1 | 0 | 0 | 0 | 1 | 0 | 0 | 0 | 0 | 1 | 0 | 0 | 0 | N/A | N/A | N/A | 1 | 1 |
| El-Tamawy et al (2019) | 1 | 1 | 0 | 0 | 0 | 1 | 1 | 0 | 0 | 1 | 1 | 1 | 0 | 0 | 0 | 1 | 0 | 0 | 0 | 0 | 1 | 0 | 0 | 0 | N/A | N/A | N/A | 1 | 1 |
| Neva et al (2019) | 1 | 1 | 0 | 0 | 1 | 1 | 1 | 1 | 1 | 1 | 1 | 1 | 0 | 0 | 1 | 1 | 1 | 0 | 1 | 0 | 1 | 0 | 1 | 1 | 1 | 1 | 1 | 1 | 1 |
| Wang et al (2019) | 1 | 1 | 0 | 0 | 0 | 0 | 1 | 1 | 1 | 1 | 1 | 1 | 1 | 1 | 0 | 1 | 1 | 0 | 1 | 0 | 1 | 0 | 1 | 0 | N/A | N/A | N/A | 1 | 1 |
| Zhang et al (2019) | 1 | 1 | 0 | 0 | 0 | 1 | 1 | 0 | 1 | 1 | 1 | 1 | 0 | 0 | 0 | 1 | 0 | 0 | 1 | 0 | 1 | 0 | 1 | 0 | N/A | N/A | N/A | 1 | 1 |
| Wang et al (2020) | 1 | 1 | 0 | 0 | 1 | 1 | 1 | 1 | 1 | 1 | 1 | 1 | 1 | 0 | 0 | 1 | 1 | 0 | 1 | 0 | 1 | 0 | 1 | 0 | N/A | N/A | N/A | 1 | 1 |
| Hassan et al(2020) | 1 | 1 | 0 | 0 | 0 | 1 | 1 | 1 | 1 | 1 | 1 | 1 | 0 | 0 | 0 | 1 | 1 | 0 | 1 | 0 | 1 | 0 | 1 | 1 | N/A | N/A | N/A | 1 | 1 |
| Ke et al (2020) | 1 | 1 | 0 | 0 | 0 | 1 | 1 | 0 | 1 | 1 | 1 | 1 | 0 | 0 | 0 | 1 | 0 | 0 | 1 | 0 | 1 | 0 | 1 | 0 | N/A | N/A | N/A | 1 | 1 |
| Gong et al (2021) | 1 | 1 | 0 | 0 | 0 | 1 | 1 | 0 | 0 | 0 | 0 | 0 | 0 | 0 | 0 | 1 | 0 | 0 | 0 | 0 | 0 | 0 | 0 | 0 | N/A | N/A | N/A | 0 | 0 |
| Note: 1 = reported. 0 = not reported. | | | | | | | | | | | | | | | | | | | | | | | | | | | | | |

**Fig. S1** The funnel plot for the meta-analysis regarding the rMT of the affected M1 after multiples session of low frequency rTMS to the unaffected M1.

**Fig. S2** The funnel plot for the meta-analysis regarding the rMT of the unaffected M1 after multiple sessions of low frequency rTMS to the unaffected M1.

**Fig. S3** The funnel plot for the meta-analysis regarding the aMT of the unaffected M1 after multiple sessions of low frequency rTMS to the unaffected M1.

**Fig. S4** The funnel plot for the meta-analysis regarding the MEPs of the affected M1 after a single session of low frequency rTMS to the unaffected M1.

**Fig. S5** The funnel plot for the meta-analysis regarding the MEPs of the unaffected M1 after a single session of low frequency rTMS to the unaffected M1.

**Fig. S6** The funnel plot for the meta-analysis regarding the MEPs of the affected M1 after multiple sessions of low frequency rTMS to the unaffected M1.

**Fig. S7** The funnel plot for the meta-analysis regarding the MEPs of the unaffected M1 after multiple sessions of low frequency rTMS to the unaffected M1.

**Fig. S8** The funnel plot for the meta-analysis regarding the rMT of the affected M1 after multiple sessions of high frequency rTMS to the affected M1.

**
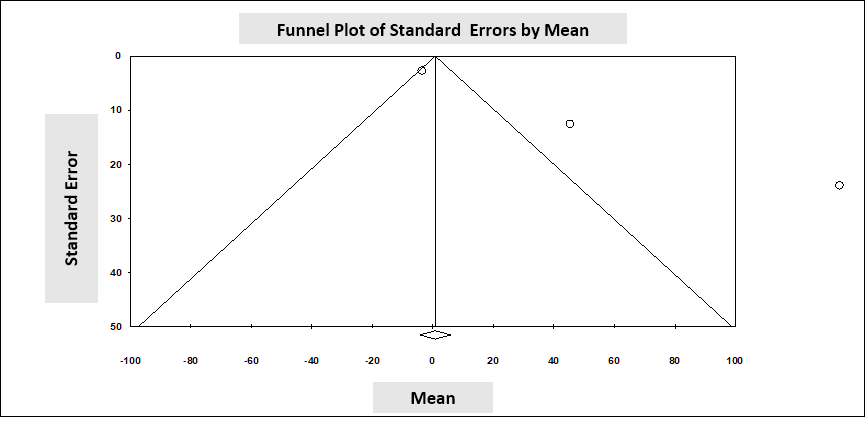
**

**Fig. S9** The funnel plot for the meta-analysis regarding the MEPs of the affected M1 after a single session of high frequency rTMS to the affected M1.

**Fig. S10** The funnel plot for the meta-analysis regarding the MEPs of the affected M1 after multiple sessions of high frequency rTMS to the affected M1.

**Fig. S11** The funnel plot for the meta-analysis regarding the MEPs of the unaffected M1 after multiple sessions of high frequency rTMS to the affected M1.

**Fig. S12** The funnel plot for the meta-analysis regarding the aMT of the affected M1 after a single session of iTBS to the affected M1.

**Fig. S13** The funnel plot for the meta-analysis regarding the aMT of the unaffected M1 after a single session of iTBS to the affected M1.

**Fig. S14** The funnel plot for the meta-analysis regarding the rMT of the affected M1 after a single session of iTBS to the affected M1.

**Fig. S15** The funnel plot for the meta-analysis regarding the rMT of the unaffected M1 after a single session of iTBS to the affected M1.

**Fig. S16** The funnel plot for the meta-analysis regarding the MEPs of the affected M1 after a single session of iTBS to the affected M1.

**Fig. S17** The funnel plot for the meta-analysis regarding the MEPs of the unaffected M1 after a single session of iTBS to the affected M1.
